# Visible Light Communications-Based Assistance System for the Blind and Visually Impaired: Design, Implementation, and Intensive Experimental Evaluation in a Real-Life Situation

**DOI:** 10.3390/s23239406

**Published:** 2023-11-25

**Authors:** Alin-Mihai Căilean, Sebastian-Andrei Avătămăniței, Cătălin Beguni, Eduard Zadobrischi, Mihai Dimian, Valentin Popa

**Affiliations:** 1 Integrated Center for Research, Development and Innovation in Advanced Materials, Nanotechnologies and Distributed Systems for Fabrication and Control, Stefan cel Mare University of Suceava, 720229 Suceava, Romania; sebastian.avatamanitei@usm.ro (S.-A.A.); catalin.beguni@usm.ro (C.B.); eduard.zadobrischi@usm.ro (E.Z.); dimian@usm.ro (M.D.); valentin@eed.usv.ro (V.P.); 2 Department of Computers, Electronics and Automation, Stefan cel Mare University of Suceava, 720229 Suceava, Romania

**Keywords:** blind person assistance, light dimming, visually impaired assistance solutions, visually impaired person, visible light communications, wearable devices, wearable sensors

## Abstract

Severe visual impairment and blindness significantly affect a person’s quality of life, leading sometimes to social anxiety. Nevertheless, instead of concentrating on a person’s inability, we could focus on their capacities and on their other senses, which in many cases are more developed. On the other hand, the technical evolution that we are witnessing is able to provide practical means that can reduce the effects that blindness and severe visual impairment have on a person’s life. In this context, this article proposes a novel wearable solution that has the potential to significantly improve blind person’s quality of life by providing personal assistance with the help of Visible Light Communications (VLC) technology. To prevent the wearable device from drawing attention and to not further emphasize the user’s deficiency, the prototype has been integrated into a smart backpack that has multiple functions, from localization to obstacle detection. To demonstrate the viability of the concept, the prototype has been evaluated in a complex scenario where it is used to receive the location of a certain object and to safely travel towards it. The experimental results have: i. confirmed the prototype’s ability to receive data at a Bit-Error Rate (BER) lower than 10^−7^; ii. established the prototype’s ability to provide support for a 3 m radius around a standard 65 × 65 cm luminaire; iii. demonstrated the concept’s compatibility with light dimming in the 1–99% interval while maintaining the low BER; and, most importantly, iv. proved that the use of the concept can enable a person to obtain information and guidance, enabling safer and faster way of traveling to a certain unknown location. As far as we know, this work is the first one to report the implementation and the experimental evaluation of such a concept.

## 1. Introduction

According to current statistics, there are about 43.3 million blind people worldwide [1], counting for about 0.48% of the total human population. Although in the last two decades, modern medicine had increased success in healing blindness, some of the current studies estimate that in the next 30 years, the number of blind people will increase by up to three times due to the growing and aging population [2]. As one can imagine, blindness has a major impact on a persons’ life, from social integration to an increased susceptibility to accidents. For instance, the unemployment rate among blind individuals is three times higher than the average, whereas the risks associated with navigating sidewalks are at least twice as high [1,2]. Moreover, in the case of young people, blindness restricts the access to normal education and limits personal progress.

If it is to define blindness, one of the most basic and simple definitions would be as an inability to see, caused by an incapacity to discern light from darkness. In this context, blind people “see” the world through their other senses, most often by hearing and touching. Thus, blindness and severe visual impairment disturb a person’s capacity to receive visual information and can have various causes, including congenital conditions, eye injuries, diseases, or degenerative conditions.

The development of new solutions to assist blind people is a major research domain having great potential to enhance the daily lives of such people [3,4,5,6]. These innovative solutions aim to augment the perception of the surrounding environment for the blind and severely visually impaired. Since a Visually Impaired Person (VIP) cannot rely on their sight, these systems must possess the capability to sense the surroundings, identify pertinent information, and convey it to the user through their other senses, with hearing and touch being the most suitable sensory channels for this purpose. To effectively perceive the environment, these devices incorporate various arrays of sensors, including ultrasound sensors, Passive InfraRed (PIR) sensors, Inertial Measurement Unit (IMU) sensors, LiDAR, GPS, and/or cameras. Once the sensory data are collected, a data fusion algorithm is employed to analyze them and provide the user with information that is not only accurate but also relevant, useful, and presented in an appropriate manner. At present, some of the most advanced solutions designed to assist VIPs are based on Artificial Intelligence (AI), specifically using neural networks for tasks like computer vision and data analysis [6]. These AI-driven systems play a crucial role in processing sensory input and providing meaningful insights to help individuals with visual impairments navigate and interact with their surroundings more effectively.

In the context of addressing the needs of visually impaired individuals, this article introduces an innovative concept aimed to provide assistance discreetly and effectively. To ensure a subtle and inconspicuous design, this concept takes the form of a backpack equipped with a range of smart features. The primary function of this backpack couples the potential of the Visible Light Communications (VLCs) technology [7,8], to facilitate information transmission to the user through the indoor lighting systems. It can be recalled here that VLCs are a new wireless communication technology that uses the visible light for simultaneous illumination and data transfer [7,8], with high potential in distance measurement, relative positioning [9], and environment sensing [10]. Consequently, different from other technologies, VLC is developing on top of a preexisting LED lighting infrastructure, providing it with a high potential to be a ubiquitous technology. In addition to the VLC features, for situations where outdoor conditions prevail and/or when VLC coverage is unavailable, the prototype incorporates additional sensors designed for obstacle detection. These sensors offer a wide total coverage angle of 240 degrees, primarily focused on the front of the user, enhancing safety and awareness. Once the relevant information is obtained from the VLC infrastructure through the VLC receivers and/or from the obstacle detection sensors, the system is able to dispatch it for the user by audio and/or by haptic means. Therefore, this article aims to provide the findings from the experimental evaluation of the VLC-based smart backpack within a complex real-world scenario. In this scenario, a visually impaired user intends to navigate from point A to point B, relying on guidance provided by the backpack. The results of this evaluation are highly promising, as they show that the prototype enables the user to receive crucial information and subsequently reach their desired destination efficiently, discreetly, swiftly, and most importantly, in a secure manner. Thus, this article continues and complements [11,12], by providing a comprehensive presentation of the prototype design, the implementation process, and additional experimental results confirm the benefits of the proposed concept.

The rest of this article is structured as follows. Section 2 provides a brief overview concerning the trends in developing blind persons-assisting solutions and a motivation for the use of the VLC technology in such applications. Then, Section 3 debates the aspects regarding the design and implementation of the VLC-based blind persons’ assistance solution, presenting the requirements of the system and describing the manner in which this system responds to them. Next, once the prototype has been presented, Section 4 comes to deliver the results of the experimental evaluation of the system’s components as well as the result of the concept evaluation in a complex user-assistance situation. Then, Section 5 provides a discussion meant to point out the importance of this work and of its possible perspectives, and Section 6 provides the conclusions of this article.

## 2. State-of-the-Art Systems and Solutions for Visually Impaired Persons Assistance and Visible Light Communications Potential

### 2.1. Existing Solutions and Approaches in Visually Impaired Persons’ Assistance

The development of assistance solutions for blind and severely visually impaired people implies several challenges. Thus, in order to efficiently guide the VIP, the assistance solution must be able to identify the location of the user. Secondly, the solution must be able to identify the relevant information that should be useful to the user, and thirdly, it should be able to deliver the information in an adequate manner [13].

Generally, users’ location can be established with the help of various wireless communication technologies, such as Bluetooth [14], or Wi-Fi, based on computer vision and inertial sensing [15], or with the help of the smartphone camera [16], while providing positioning errors between 0.4 to 1.5 m. To identify the information from the area, camera-based image navigation solutions are widely used [17,18]. These solutions imply a camera and specialized software that is able to recognize objects from the scene. Recently, various other solutions, such as artificial intelligence and Computer Vision (CV) applications, are swiftly progressing [19,20]. For relative positioning and distance measurement, as well as for obstacle detection, LIDAR, ultrasound and camera recognition systems are also used [21]. Next, once the relevant information is identified, it is transmitted to the user as audio information or by haptic means.

In terms of user solutions, blind assistance solutions are generally integrated into various devices, but most often into smart glasses and smart canes, while also being developed as personal computer or smartphone-compatible applications. These technologies are designed to enhance daily lives of individuals with visual impairments by providing real-time assistance and information. These smart devices and applications offer features like persons and object recognition, navigation aids, and text-to-speech capabilities, allowing users to receive auditory or haptic feedback about their surroundings. Smart canes are frequently equipped with sensors and GPS for obstacle detection and navigation support [22], Computer and smartphone applications offer accessibility features such as voice assistants, screen readers, and GPS-based navigation. These integrated software solutions aim to improve mobility, independence, and overall quality of life for VIPs. The high prevalence of personal computers and smartphones has led to the development of multiple software applications that are meant to assist visually impaired persons. Some of these applications are briefly presented in Section 2.2.

### 2.2. Commercial Software Applications for Blind and Severely Visually Impaired Persons’ Assistance

A significant part of software applications designed for individuals with visual impairment is closely related to assistive technology, playing a crucial role in enabling voice-based reading. Their main purpose is to convert text into speech, making it more accessible for the blind and visually impaired and facilitating their access to written information, documents, and websites. Popular software applications like Job Access with Speech (JAWS) [23] and NonVisual Desktop Access (NVDA) [24] are widely used for this purpose. JAWS, developed by Freedom Scientific, is renowned for its ability to provide voice-based reading and enhanced accessibility, converting on-screen text and graphics into speech or Braille. Its features include compatibility with online platforms, the Microsoft Office suite, web browsers, email applications, and social media. NVDA, on the other hand, is an open-source software solution that offers similar functionalities but stands out for its portability and compatibility across various operating systems and digital resources.

Additionally, there are electronic Braille devices, GPS navigation systems integrated with mobile applications like Lazarillo [25] and BlindSquare [26], voice recognition systems like Siri [27] and Google Assistant [28], or VoiceOver [29] for Apple devices, which provide accessibility features and support for users with visual impairment. There are also mobile applications designed to identify colors in the user’s surroundings, such as Be My Eyes [30], which allows users to request assistance from volunteers. These assistive technologies, along with handwriting-to-text conversion apps and Braille printers, contribute to creating a more inclusive and accessible environment for individuals with visual impairments.

Considering the important role of education, as well as the fundamental right to education, assistance software solutions for this purpose have been developed. Thus, specialized educational resources, AI assistance in online education, wearable technologies for content recognition and vocal feedback, and Haptic Wearables for urban navigation have been developed to enhance the daily lives and educational experiences of people with visual impairments. The rapid advancement of technology, including Virtual Reality (VR) and Augmented Reality (AR), has revolutionized the field of assistive technologies, providing innovative and intuitive solutions for the visually impaired. The integration of emerging technologies is transforming the way individuals with visual impairments access information and engage in educational processes. Accessibility standards like Web Content Accessibility Guidelines (WCAG) [31] have played a pivotal role in ensuring that digital learning platforms and online courses are inclusive for individuals with disabilities.

While current studies and developments in this field are still in their early stages, the complexity of addressing the needs of VIPs has led to significant advancements, particularly in the medical field. These advancements range from retinal implants to facial recognition, text reading, and audio playback, with the ultimate goal of enhancing the quality of life for individuals with visual impairment.

### 2.3. Visible Light Communications and Their Potential in Blind and Severely Visually Impaired Persons’ Assistance

Over the past decade, VLC has emerged as an exciting wireless technology that has witnessed significant advancements. As previously mentioned, VLC uses visible light not only for illumination but also as means of transmitting data, thereby enabling pervasive wireless communication. Consequently, VLC has the remarkable capability to transform any LED light source into a data transmission device. Moreover, extensive research efforts have unlocked the potential of VLC for achieving highly precise localization, making it a valuable technology for delivering position-specific data. In contrast to traditional Radio Frequency (RF) communication, where a central device covers a wide area, VLC networks exploit the inherent properties of light. These characteristics allow for the deployment of a multitude of optical access points and the enhancement of overall performance.

Considering that LED lighting systems are an integral part of our daily lives, serving not only to illuminate our surroundings but also to provide essential visual information, it becomes apparent that their ubiquitous presence can be harnessed for a broader spectrum of applications. In this context, VIPs can derive substantial benefits from VLC’s extensive coverage and its capacity to offer location-specific data. Thus, one can see that the VLC technology has the intrinsic means to solve a significant part of the tasks mentioned in Section 2.1, as it has the potential to identify users’ locations and to timely deliver location specific data. Furthermore, as the VLC technology is developing on top of a preexisting and widely available lighting network, its potential is very high. On these grounds, VLC empowers users to be constantly aware of their precise location and receive pertinent information relevant to their specific surroundings.

However, despite the promising potential of VLC in assisting visually impaired individuals, there remains a relative lack of relevant research focused on practical demonstrations of these concepts. More concerted efforts in this area are needed to fully explore and use the capabilities of VLC technology for the benefit of those with visual impairments. Examples of preliminary works focused on VLC use in blind persons’ assistance can be found in [32,33,34,35]. Although these works emphasize the benefits of VLC and its compliance with visually impaired assistance, their implementation is still at a low Technology Readiness Level (TRL), whereas the experimental results are far from being relevant for real-life utilization. On the other hand, these works have the merit of pushing things forward in the right direction.

## 3. Conceptualization and Implementation of the Visible Light Communications-Based Smart Backpack for Blind and Severely Visually Impaired Persons’ Assistance

This section approaches the issues related to the design and the practical implementation of the VLC-based blind persons’ assistance smart backpack prototype. It aims to provide the purpose, the development guidelines, their argumentation and to illustrate the transition from design guidelines to experimental prototype.

### 3.1. Prototype Conceptualization

#### 3.1.1. Purpose Statement

The existing blind-assistance literature and the market segment oriented towards their guidance enables the identification of several types of assistance applications, from solutions that help a person to read information [23,36] to software solutions that help a person to fully use a computer and its applications [26,30]. In this context, the purpose of the proposed solution is to provide blind and severely visually impaired persons with information that enables them to navigate in unfamiliar places based on personal guidance and user location specific information. Furthermore, in order to optimize the path, as well as to minimize the risks associated with the movement in unfamiliar locations, the proposed solution aims to be able to detect possible obstacles (i.e., open doors, boxes, chairs, dispensers etc.) in users’ paths and to warn them. Another problem that the system aims to address is related to the blind person’s ability to maintain a straight direction when it is necessary. Consequently, the system targets to monitor and to provide the users with information about their path and trajectory.

#### 3.1.2. Visible Light Communications-Based Smart Backpack for Visually Impaired Persons’ Assistance: Requirements and Guidelines

***From users’ perspective***, in order to be effective, a human assistance solution should be *useful* to its users, *versatile and simple to use*, should have a high *users’ acceptance*, and should be *cost-efficient*. These are the preliminary requirements that have been imposed for the blind assistance solution.

*Usefulness:* In order to be useful for blind persons’ assistance, a system should be able to provide its users with information that they are not able to perceive through their sight. Nevertheless, as the environment has a lot of visual information to offer, a system should be able to analyze the available information and should be able to offer only the relevant information. Otherwise, too many data can distract the user’s attention, making the system less useful. This implies a careful analysis of the available information and an adequate consideration of the users’ needs.

*Use simplicity and versatility:* The system should be able to translate visual information into other types of sensorial data, whereas in this case, hearing and touching/sensing are the most straightforward senses that could be used. Nevertheless, as sometimes blind users already rely on their hearing in order to perceive the environment, the system should be able to provide users the possibility to use hearing options only when they want. Another aspect regarding versatility is related to a system’s ability to provide additional services and/or functionalities. For example, many successful blind-assistance solutions are applications that can be installed on a smartphone. Their success is based on the fact that the blind assistance function can be integrated on an already useful device, which is now able to provide an additional function. Another aspect related to versatility is related to a solution’s ability to remain helpful in as many situations as possible. For example, many GPS-based blind guidance solutions become useless in situations with no GPS coverage, which represents a major issue.

*Users’ acceptance:* Many VIP support solutions encounter a common challenge related to their hardware design. To enhance the functionality, numerous sensors are often incorporated. While this approach improves the perception of the user’s surroundings, it frequently results in final prototypes that are overly sized and lack aesthetic appeal, making them unaesthetic and uncomfortable for users to wear. Thus, it is obvious that wearing such a conspicuous and bulky device can inadvertently highlight the users’ impairment, thereby limiting the device’s overall practicality and benefits.

*Cost-efficiency:* Cost-efficiency is a feature that can be attributed to a system that is not necessarily cheap, but whose benefits are high with respect to the cost. Therefore, adding extra features to a product can make it cost-efficient if multiple users’ needs are satisfied.

***From a developer’s perspective***, the Visible Light Communications technology imposes a series of constraints. As previously mentioned, VLC assumes the use of the LED lighting equipment for simultaneous illumination and data transmission.

*Data transmission as a secondary feature:* Because the digital information sent through an existing lighting system is a secondary feature, it must not affect the primary function, as an illumination device, in any way. Therefore, the use of the VLC technology in the blind-assistance purpose should respect the following principles:

- VLC should not impact the existing lighting infrastructure from a hardware point of view or should have a minimum impact;
- VLC function should not affect lighting from a regular user visibility point of view, meaning that the same lighting intensity should be provided; thus, light intensity should not be increased in order to improve the Signal-to-Noise Ratio (SNR), nor it should be decreased unnecessarily;
- VLC should not generate visible or perceivable flickering;
- When light dimming is necessary, data transmission should be available.

*Communication coverage*: Another aspect that should be considered is related to the coverage area. In order to be useful and safe, the VLC system should have the potential to provide wide area coverage. Therefore, while considering the existing distribution of the lighting devices within the space, the prototype should be able to ensure VLC data transmission for the entire envisioned area. 

*User-centered data distribution:* In order to be effective in blind user assistance, the solution should be able to localize the user, in order to transmit location specific information. Nevertheless, different from IoT applications or from robot control applications where centimeter precision is required, in this case, high precision localization is less important, as the main objective is to have an estimation of the user’s position.

### 3.2. Visible Light Communications-Based Smart Backpack for Visually Impaired Persons: Implementation Process

**Figure 1:**
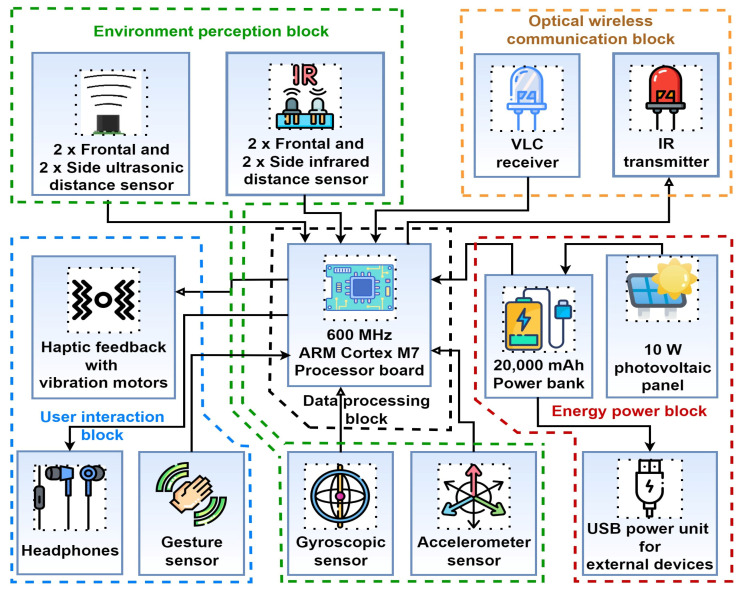
Schematic representation of the VLC-based smart backpack for blind users’ assistance.

Within the upper-described context, this ongoing project was initiated with the purpose of designing, developing, and experimentally testing a novel visually impaired and blind assistance device that is *discreet*, *versatile*, *user-friendly,* and *cost-efficient*. The main aim of the project is to create a device that seamlessly integrates multiple functions into an item that does not draw attention to the user’s impairment. To achieve this goal, the proposed concept takes the form of an everyday backpack, making it useful already. This design approach offers ample space for concealing the various sensors required for enhanced functionality, ensuring that the final product supports the aforementioned requirements of discretion, utility, and multi-purpose use. The schematic of the proposed design is illustrated in Figure 1. As one can see, in addition to the basic backpack, the system consists of five main blocks.

The first block is the *energy power block*. For improved utility and versatility, the concept uses a 10 W PhotoVoltaic (PV) panel that has the purpose of improving the energy-efficiency and the autonomy of the system. The resulting electricity/energy is stored in a 5V/20,000 mAh power bank, which is used to power the smart backpack’s other blocks. However, for improved utility, the user can also recharge thier personal devices, such as smartphones or smartwatches, through several USB ports.

**Figure 2:**
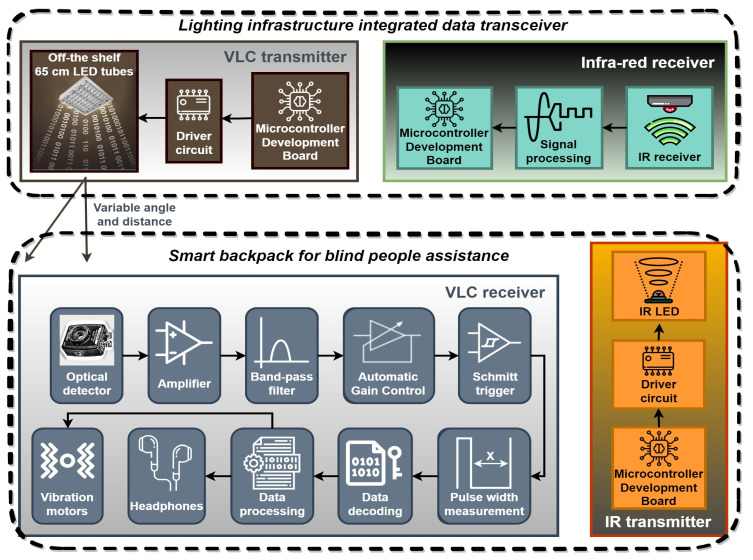
Schematic of the smart backpack visible light communications component.

The most important component of the concept is the *optical wireless communications block*. To facilitate data exchange with the indoor lighting system, the smart backpack prototype employs two optical transceivers positioned on the upper side, more precisely on the backpack braces. On the other side of the VLC channel, the infrastructure-integrated wireless communication component includes an optical transceiver fitted into the indoor lighting network. These transceivers employ visible light for receiving data from the indoor lighting system and Infrared (IR) links for uploading information requests. The schematic of the prototype’s optical wireless communications component is shown in Figure 2, emphasizing the infrastructure-integrated transmitter and one of the smart backpack transceivers. This bidirectional connection allows the user to request information and, in future developments, can potentially enable the VLC lighting infrastructure to determine the user’s location using Time-of-Flight (ToF) measurement properties combined with Received Signal Strength protocols [9]. Currently, user requests primarily focus on emergency assistance, and are limited to several specific points of interest such as the location of restroom, elevator, door, stairs, campus restaurant, or a few other objects. Nevertheless, as the concept is further developed, additional points of interest could be defined.

The VLC transmitter has been integrated into the indoor lighting infrastructure from one of our research laboratories. Specifically, the actual setup is based on a luminaire positioned at a height of 3.5 m above the floor. The luminaire uses four 60 cm off-the-shelf LED tubes, each having a power of 9 W. Controlled through a digital driver by an ARM Cortex M7 processor operating at 600 MHz, the LED tubes-based luminaire becomes an information broadcasting device. For VLC data transmission, the system uses an adapted form of Variable Pulse Position Modulation (VPPM) [37], with asynchronous communication, capable of providing a data rate of up to 100 kb/s. VPPM is a modulation technique suitable for VLC applications, which combines Pulse Width Modulation (PWM) and Pulse Position Modulation (PPM), enabling precise light dimming without compromising data transmission. The design provides optical intensity of 101 lux at the workspace level (2.7 m from the ceiling), when a 50% duty cycle is used. However, the light intensity can be adjusted from virtually the off state (1% duty cycle) to the nearly maximum intensity of the lighting device (obtained at a 99% duty cycle). Thus, it is important to emphasize that the system is designed to be compatible with light dimming, making it suitable for energy-saving applications while maintaining its blind assistance functions. For situations that imply high optical noise, the concept is also compatible with Binary Frequency-Shift Keying (BFSK) modulation [38], solution that further enhances the system’s resilience to noise.

In its turn, the VLC receiver represents a vital component of the smart backpack. It consists of an optical collecting system, a signal processing segment, and a data processing unit. The front-end employs an optical filter used to eliminate unwanted spectral components and a PIN photodiode-based optical detector having a Field-of-View (FoV) of ±53°, which is able to convert the incident light into a proportional electrical signal. Then, the signal processing module handles tasks such as signal band-pass filtering, signal amplification, and signal reconstruction. More precisely, the signal passes through a 1 kHz–500 kHz 4th order band-pass Bessel filter, several preamplification stages, an adaptive gain control circuit that stabilizes the signal amplitude, and a Schmitt trigger circuit which provides the digital output containing the binary data. Finally, the data processing unit is responsible for real-time data decoding, data analysis, and Bit Error Rate (BER) measurement. To accomplish these tasks, an ARM M7 Cortex processor running at 1008 MHz is used. Table 1 summarizes the parameters of the prototype’s VLC component.

**Figure 3:**
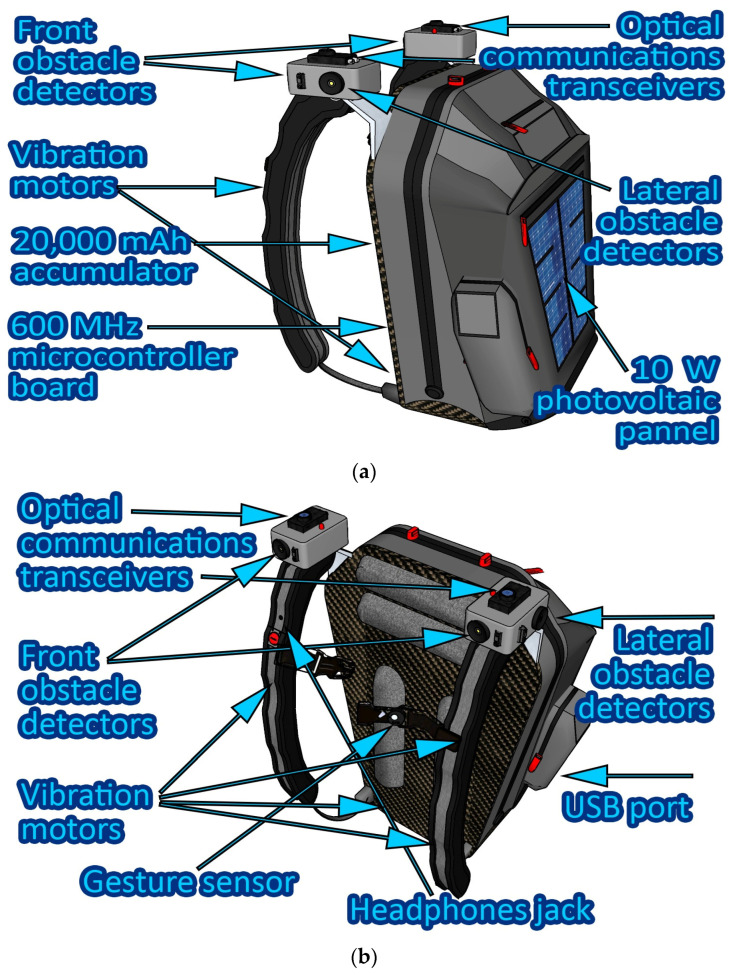
Visible light communications-based smart backpack for blind and severely visually impaired persons’ assistance: (**a**) Front and lateral side view; (**b**) Backside view.

The following block is the *environment perception block*. This unit consists of sensors that have the purpose of analyzing the users’ movement and to detect potential obstacles. For improved effectiveness, an array of four obstacle-detection modules is distributed on the backpack. Two of these modules are positioned on each lateral side, while the other two are oriented towards the front, as depicted in Figure 3. Within each detection module, there resides a combination of a PIR sensor and an ultrasound sensor. The inclusion of these two distinct sensor types is motivated by their high compatibility with each other, which serves to reinforce the reliability of the information provided to the user. These obstacle-detection modules are strategically placed, each encompassing a 60-degree angle, resulting in a 240-degree coverage. Each module has a detection range set at 90 cm. Although longer ranges up to 250 cm could be used, it was considered that the most relevant information is the one from the immediate vicinity. Choosing a wider range can lead instead to situations in which all the sensors are constantly detecting certain obstacles, providing the user with too much information, which in turn can be rather distracting and less effective for the user. Anyway, the obstacle detection range can be further increased up to more than 250 cm if the user requires it. The obstacle detection modules are also vertically adjustable ensuring adaptability to the user’s specific needs. The core function of these modules is to identify and alert the user regarding the presence of objects or individuals obstructing their path, thereby enhancing safety and facilitating smooth navigation. The environment perception unit also englobes a gyroscopic sensor and an accelerometer. The gyroscopic sensor is used to monitor users’ orientation, in order to properly guide them in the right direction. As the chances of falling are significantly higher for blind persons, the accelerometer is envisioned to identify potential situations in which users’ have fallen and/or are injured.

As one can see, while the communication component excels in indoor VLC-covered areas, the obstacle-detection component complements it by providing supplementary information. More importantly, it also extends its utility beyond the limits of VLC-enabled indoor environments, making it useful for both indoor and outdoor settings. Thus, once the smart backpack prototype has received the VLC data from the indoor lighting infrastructure and the information from the environment perception module, a data processing unit (again an ARM Cortex M7 at 600 MHz board) analyzes it and decides what type of information should be transmitted to the user.

Another important module of the smart backpack prototype consists in the block that enables the user to exchange information with the backpack and vice versa. Thus, to request information on certain points of interest, the user uses a gesture sensor which recognizes predefined gestures and assigns them to a certain request. The request is then analyzed and communicated through the IR transmitter embedded module to the optical transceiver integrated in the indoor lighting infrastructure. The lighting infrastructure module analyzes the request and provides a response which is transmitted using VLC. When the backpack VLC component receives the data, it analyzes it and provides the user with the requested information. The interaction with the user is made through a synthesized voice. Nevertheless, there are situations in which the users might require their hearing for other purposes. For such circumstances, the smart backpack integrates a series of four vibration motors. Two of them are located on the backpack shoulder strap, one for each arm, and the other two are located on the bottom of the backpack, being in contact with the user’s lobar area, left side, and right side. Thus, based on a predefined haptic language that the user has to get used with, information is transmitted. The four vibration motors are also assigned to the four obstacle sensors. More exactly, each obstacle detection sensor, monitoring a certain area around the user, is assigned to one vibration motor. Thus, when an obstacle is detected on the user’s front left side (i.e., the user could hit the obstacle with his left shoulder as he moves forward), the vibration motor on the left shoulder strap begins to vibrate (i.e., distance dependent vibrations). Similarly, to suggest that a wall is located on the left side, the vibration motor in contact with the user’s lumbar area is activated (i.e., distance dependent vibrations). In accordance, the 90 cm monitoring range of each sensor is divided in three sectors: 0–30 cm, 30–60 cm, and 60–90 cm. Further on, when an obstacle is detected in a certain area, the vibration motor associated with that sensor generates vibrations of certain frequencies, where vibration frequency is in accordance with the distance toward the obstacle. Thus, when the obstacle is detected at 90 cm, the frequency of the vibrations is low, whereas as the user gets closer to the obstacle, the frequency of the vibrations increases. This versatility allows the system to accommodate a variety of scenarios and user preferences. Figure 3 illustrates the distribution of the modules on the smart backpack.

## 4. Experimental Testing Procedure, Experimental Results and Discussions concerning the Importance of This Work

The following section presents the aspects related to the blind persons’ assistance smart backpack intensive experimental testing procedure. It details the experimental evaluation method and the associated experimental results, focusing on the individual system components evaluation, as well as on the prototype’s evaluation in a complex setup.

### 4.1. Experimental Testing Procedure

**Figure 4:**
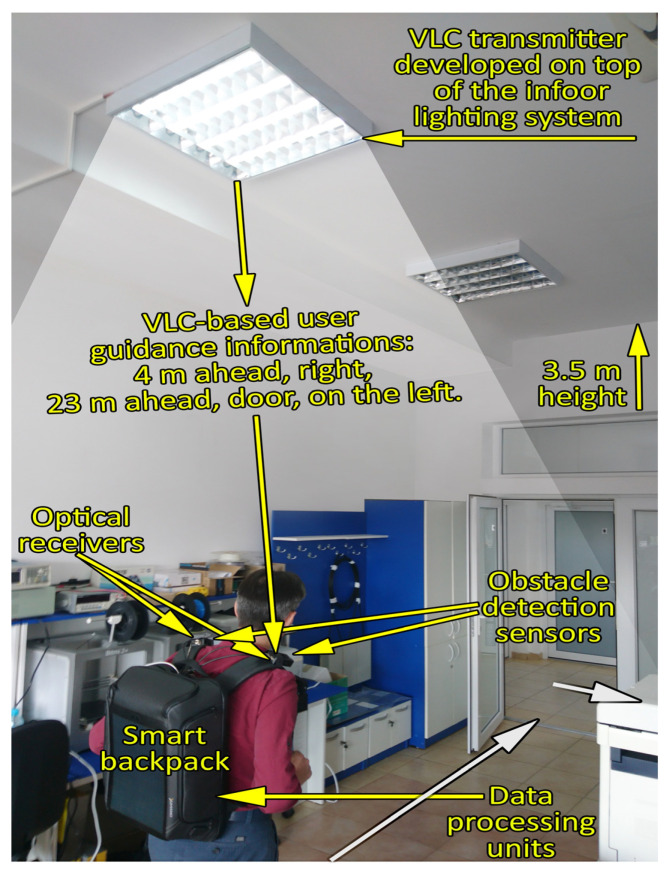
Visible light communication testing scenario: The indoor lighting system uses VLC to provide the user with information concerning a certain point of interest. After leaving the area, the user is no longer in VLC coverage and has to rely on the embedded obstacle localization sensors and on the haptic systems to travel the distance until the point of interest.

To validate the feasibility of the proposed concept and plan the future course of this project, the VLC-based smart backpack prototype underwent experimental assessment in controlled laboratory conditions. As depicted in Figure 4, the optical communications component was integrated into the indoor lighting system, forming the basis for the experimental testing environment. These initial tests were structured into two distinct phases, each serving a unique and different purpose.

In the first phase, the system’s capability to receive data from the indoor lighting component and relay it to the user in the form of audio and haptic information was evaluated. Thus, the first component of these tests was focused on investigating the VLC’s ability to provide low BER communication, to support communication in light dimming conditions, to support user mobility or to support connectivity within the area of the VLC transmitter. Therefore, these tests aimed to confirm the proper functionality of the VLC component.

The second phase is focused on assessing the system’s ability to operate in scenarios where VLC coverage is unavailable or obstructed. In this regard, the backpack was used by two individuals who were blindfolded. Their task was to exit the laboratory, traverse a 23 m corridor, open a door, and reach a point situated behind it to find an object placed on a table. During this process, the user wearing the backpack initially received location guidance from the indoor lighting system through VLC. Upon processing this signal, the user was provided with audio instructions such as “*Walk 4 meters to reach the door. After passing through the door, turn right and proceed down the 23-m hallway. The destination is 1 meter behind the door, on the left side.*” As the user moved beyond the VLC-covered area, he relied only on the obstacle detection sensors integrated into the backpack. As previously mentioned, these sensors should provide relevant information to the user through haptic feedback, utilizing the four vibration motors strategically positioned within the backpack. Vibration frequencies vary based on the distance to the obstacle, with higher frequencies indicating closer proximity to the obstacle.

This experimental setup was conducted with each of the two users repeating the task only five times. Although five experiments could be considered insufficient from a statistical point of view, limited tests have been made in order to prevent the users from accommodating with the trials, a fact that would influence the results. Consequently, one can consider that the results of these tests are relevant for users traveling in unfamiliar locations. For comparative purposes, the same task was also performed by a blindfolded user who did not use the smart backpack. It is important to underscore that individuals with visual impairments often face challenges in maintaining a straight direction, and therefore, the proposed solution seeks to address this specific issue by providing navigational assistance.

Apart from these tests, where the smart backpack should guide the user from one point to the other, by relying on either VLC or on obstacle detection sensors, another obstacle detection challenge has been introduced. In order to test the prototype’s effectiveness in providing a safe path, obstacles were intermittently introduced along the path to test if the device could assist the users in safely navigating from one location to another. This third test aimed to determine if the prototype is able to prevent users from bumping into persons present in the path, or into certain objects. Based on a meeting with a blind student and on studies focusing on blind persons’ problems, our team managed to find that one of the most challenging situations is when dealing with obstacles that are located at chest and head level, as these objects cannot be identified with the help of the blind stick. Otherwise, the blind stick is very effective in locating objects and ground level anomalies.

### 4.2. Experimental Results

#### 4.2.1. Experimental Results for the Visible Light Communications Component Evaluation

One of the purposes of the experimental evaluation of the VLC component was to confirm the capacity of the system to reliably transmit data from the LED-based luminaire VLC transmitter to the VLC receiver. Compared to the vehicular VLC channel, which is primarily in outdoor conditions [37,38,39,40], the indoor one is definitely less challenging, as it involves only a few meters’ communication range, less optical interferences, and a smaller degree of unpredictability, so good results would be expected.

Another purpose of this experimental investigation is to determine the system’s ability to maintain the connectivity when the user (i.e., the VLC receiver) is moving inside the VLC transmitter area. To be effective, such a system should be able to maintain the connectivity even within a wider area, and not only under the luminaire. More precisely, as the VLC receiver is upwards oriented, when it is moving away from the VLC transmitter, the incidence angle is increasing. Nevertheless, as the current generated by the photodiode has an incident angle cosine dependency, whereas the luminaire has a light direction oriented downwards, toward the workspace, one can see that the communication link can be affected. Therefore, these experiments are also useful in determining the system’s communication coverage for a given BER limit.

**Figure 5:**
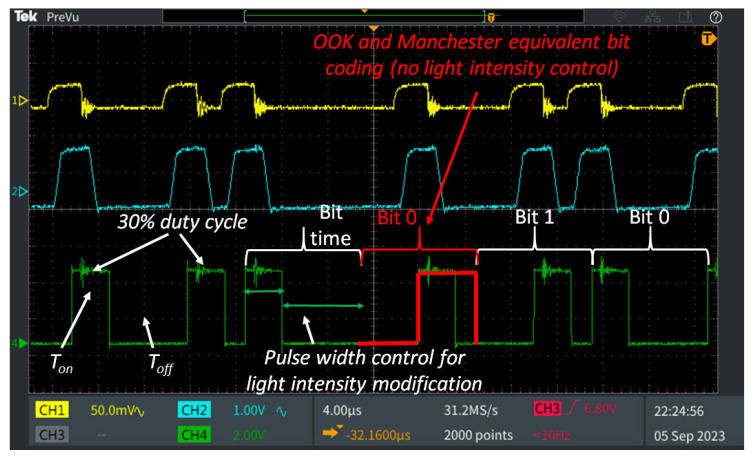
Illustration of signal processing at the VLC receiver level: Channel 1 (yellow) represents the output of the optical receiver; Channel 2 (cyan) represents the signal after filtering and amplification; Channel 4 (green) shows the digital signal that is used by the microcontroller to extract the binary data. The figure also depicts the use of the VPPM modulation in a 30% duty cycle example, the light dimming principles, and provides a comparison with the traditional Manchester coding.

Thirdly, the purpose of these experiments is to confirm the VLC system’s ability to provide simultaneous light dimming and data communication. As energy efficiency is becoming a major preoccupation for human society, the compatibility with light dimming or the system’s ability to work in situations in which the user does not need the lighting function become very important. Figure 5 exemplifies an oscilloscope capture showing the signals received by the VLC receiver. It also displays the manner in which the incident light is gradually transformed into a ready to use digital signal. Additionally, this figure illustrates the VPPM light dimming mechanism with its working principles and a brief comparison with the classical Manchester coding. As one can see, VPPM enables a wide control over the *lights-on* period, and therefore, a good control over the light intensity. Additionally, as the *T_ON_* is similar for bit 1 and bit 0, no light flickering is introduced. The summary of the experimental results resuming the VLC component performance is provided in Table 2. As expected, the high SNR corroborated with an optimized VLC hardware design enabled the system to provide and maintain an extremely low BER. Thus, a BER lower than 10^−7^ is achieved without any use of error correcting techniques. For comparison, previous experience has shown that with an adequate hardware design and with an optimized software data extraction algorithm, a low BER can be maintained as long as the SNR does not get below a 0–1 dB limit [38,39,40]. The very low BER is also the result of the integration of an automatic gain control circuit within the VLC receiver. The AGC is constantly adjusting the signal amplification, compensating the decrease in the incident power, while also preventing over-amplification that could lead to signal distortion and in turn, to bit errors. Furthermore, the results of the intensive experimental evaluation showed that the VLC receiver is able to maintain this low BER even in light-dimming conditions, as well as in the situation in which the VLC receiver is moving away from the VLC transmitter center coverage area. Thus, it has been demonstrated that the system can provide connectivity for a circular region that can have a 3 m radius. However, as in the experimental setup, the lighting fixtures are placed 1.5 m away from each other: this demonstrates that the proposed design is more than suitable to ensure the coverage of the entire area, and that it can definitely be replicated for other areas as well. Another aspect that has to be debated is related to the systems’ ability to work in “*lights-off*” conditions. On this topic, it has been experimentally demonstrated that the VLC transmitter can work with duty-cycles as low as 1%, which generate an illuminance that can be considered close to the point where the lights are off. In its turn, it has been demonstrated that both the hardware and the software components of the VLC receiver are able to handle the 1% duty cycles. Thus, this demonstrates that the proposed concept is compatible with energy-saving applications as well as with applications where lighting is not necessary all the time. On the other hand, with duty cycles below 10%, the complexity of the software routines is forcing the hardware to lower its data rate to 10 kb/s instead of 100 kb/s, in order to keep the same quality of data transmission as for duty cycles of 10% and above. In this case, the higher BER limit is the result of a lower number of transmitted bits. Additionally, the purpose was also to suggest that when the duty cycle is lowered, a slightly higher BER is expected. The reason for this higher BER is related to the wider bandwidth imposed for the band-pass filtering.

#### 4.2.2. Experimental Results for the Obstacle Detection Component Evaluation

**Figure 6:**
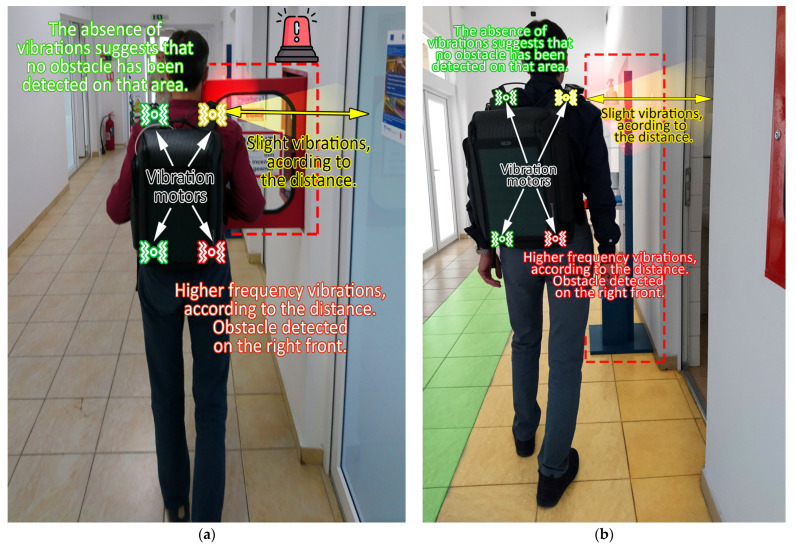
Example of smart backpack for blind persons’ assistance experimental utility: the prototype’s obstacle sensors are able to identify and inform the user that: (**a**) an open glass door is obstructing the path; (**b**) a dispenser is in the path and an open door is found on the user’s right side.

Once the performance of the VLC component was confirmed, the next phase was focused on the obstacle detection component. Before moving forward to the evaluation in a complex situation, the first tests aimed to determine if the system is able to safely localize potential dangerous objects that could obstruct the path of a blind user. As mentioned in previous sections, these tests aimed to determine the system’s efficacy in preventing the user to bump into other persons, to hit objects located in the path, and, most importantly, to localize objects that cannot be located by a blind stick, referring here to objects that are located at the chest and/or head level. The situations envisioned are illustrated in Figure 6, whereas the summary of the experimental results is provided in Table 3.

The experimental findings also demonstrate the prototype’s efficacy in assisting visually impaired individuals with key aspects of navigation and spatial awareness. Specifically, the prototype aids users in maintaining a straight trajectory, detecting the presence of individuals or obstacles in their path, signaling the proximity of a door/wall, and enhancing their overall environmental perception.

**Figure 7:**
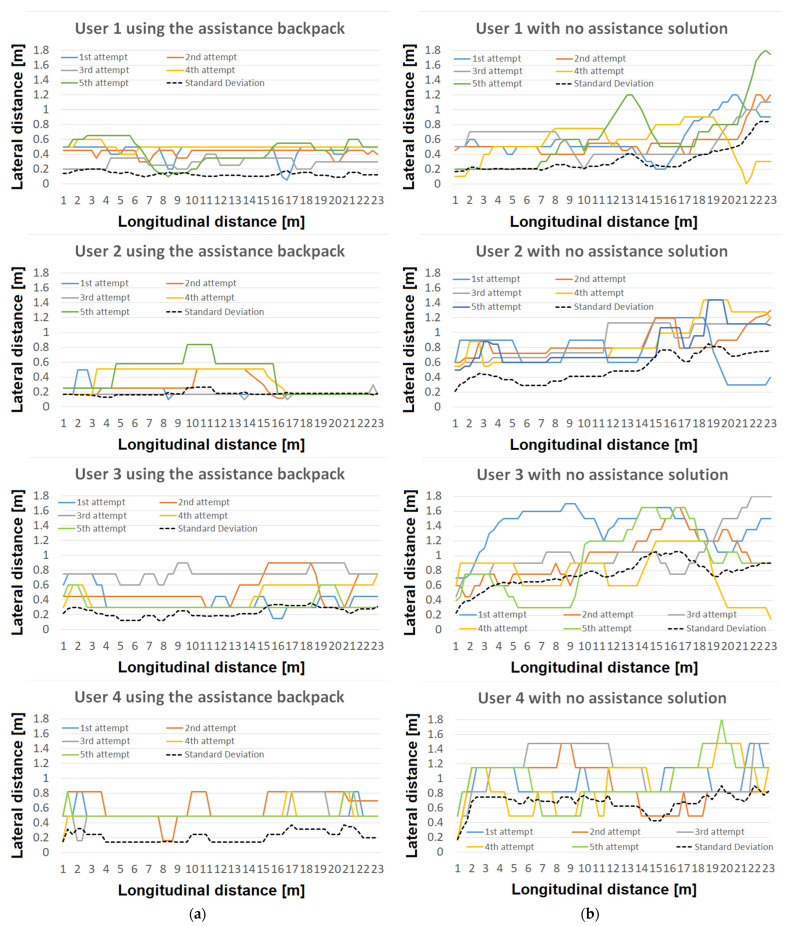
Experimental results showing the path taken by four blindfolded users, as they navigated from point A to point B while utilizing the VLC-based smart backpack. The results revealed a notable enhancement in the users’ path and a significant reduction in their travel time: (**a**) With backpack assistance; (**b**) Without any kind of assistance.

Thus, as illustrated in Figure 7, users exhibit a notably straighter trajectory when utilizing the prototype. This improved directional control, coupled with the increased confidence imparted by the system, results in a significant reduction in travel times. Consequently, users are less apprehensive about encountering obstacles, leading to a noteworthy reduction in the average travel time—from 53.6 s to 39.3 s. It is worth noting that further enhancements are anticipated as users become more accustomed to the system’s operation and as the prototype is further calibrated on users’ specific requirements, promising even greater improvements in their navigation experience.

## 5. Discussion and Future Perspectives Regarding the Use of the Visible Light Communications Technology in Visually Impaired Persons’ Assistance

Based on the results of the experimental evaluation, it can be considered that the proposed VLC-based smart backpack concept represents an innovative and versatile solution which has the potential to revolutionize navigation, communication, and safety in both indoor and outdoor environments, helping blind persons to travel in unfamiliar locations. In summary, the proposed VLC-based smart backpack offers several compelling advantages and promising prospects. The VLC technology provides the concept with a versatile data transfer solution which enables seamless data transfer in conjunction with a lighting function, making efficient use of limited power resources. Thus, the use of VLC delivers unlicensed spectrum access and a vast bandwidth of 400 terahertz, providing the support for multiple applications ranging from user localization to location-dependent data distribution. Therefore, different from most of the existing technologies, VLC is very suitable for location-oriented data distribution, providing the user with more relevant information. Accordingly, each luminaire is able to provide the user with accurate and specific information, where the information is correlated with its location, enabling an improved context-aware assistance and navigation.

One of the main advantages of VLC use in blind persons’ assistance comes from the relatively *simple integration within the preexisting lighting infrastructure*, which offers in turn the support for the development of a ubiquitous assistance solution. In addition, as the proposed solution is developing on a preexisting lighting infrastructure, the implementation cost is partially reduced. The cost-efficiency is also provided by the relatively simple architecture and by its multipurpose functionality. Thus, the cost of the solution becomes controllable, and it could be estimated that the cost of a luminaire upgrade is below 100 euros, a cost that could go even lower if mass production is adopted. Furthermore, the low energy consumption associated with LED use, VLC and light dimming functions further improves the cost-efficiency. For comparison, the operation of a RF-based solution requires continuous energy consumption. On the other hand, in VLC, the data transmission function requires no additional energy consumption as the light used for lighting purposes is also used as a carrier for the data. As lighting systems is present in all public places, from airports to schools and public institutions, the development of such a solution can provide blind persons with access and personalized support in these areas, improving their independence.

From the VLC usage point of view, the experimental results have confirmed a very low BER (i.e., ranging within 10^−7^–10^−6^), a wide area coverage, and, very importantly, the compatibility with mobility and light dimming. From this point of view, it is important to emphasize that although there are numerous works that address VLC light dimming capabilities [41,42,43,44], there are only a few that provide experimental demonstrations of such concepts [44]. Nevertheless, in [44], a limited 25–85 dimming range was demonstrated, while providing a 10^−3^ BER, and communication range below 1 m. Furthermore, demonstrating the system’s ability to maintain the data link even for a 1% duty cycle, emphasizes the concept’s compatibility with energy saving applications and with the current preoccupations for energy efficiency.

Another important contribution of this work comes from the fact that unlike most of the works that promote the development of a new notion or of a new technology, this work delivers the integration of the proposed concept into a functional device, together with an extended experimental investigation that demonstrates the concept’s utility in a relevant use case. Thus, this work not only introduces the use of the VLC technology in severely visually impaired and blind persons’ assistance, but it provides a relevant experimental demonstration for its utility. Therefore, although the proposed concept is not yet a commercial device (i.e., a TRL 10 product), it provides a TRL 6 product, providing the basis for future enhancements. Thus, this work aimed to make the transition from fundamental research to experimental research, contributing to the deployment of the VLC technology in new applications. Hence, although there are several other works that analyzed the use of VLC in blind persons’ assistance, the prototype presented in this article is one of the most advanced, benefiting from a more realistic integration, from a more intensive testing and from an overall enhanced design. Furthermore, to improve its capacity and to enable its use in areas with no VLC coverage, the smart backpack concept also integrates environment perception sensors that provide a 240° degree scanning around the user, enabling the detection of obstacles located in the users’ path. Additionally, these sensors significantly improve the trajectory and travel time of visually impaired users, instilling confidence and reducing the risk of collisions with other persons or with obstacles, reducing in turn the chances of accidents during the walk. Thus, as the experimental results have confirmed, the use of this sensor fusion enabled the user to maintain a straighter trajectory and to improve its confidence, resulting in in shorter walking times.

An important aspect that should be clarified is related to the functionality of the proposed solution in multiple lighting devices setup. From this perspective, the current version of the prototype did not address the issues associated with multiple lighting devices. Nevertheless, the indoor VLC literature has widely addressed these issues and it was able to find the proper solutions that allow user mobility, provide the hand-over mechanisms between multiple light sources, and enable multiple-users resource sharing [45,46,47]. Consequently, it can be considered that user movement and the synchronization between multiple lighting devices are not that problematic. One limitation of the proposed solution comes from the relatively low data rates. For comparison, record data rates in indoor VLC applications go up to a few tens of Gb/s [48]. Although the experimental results showed that the prototype is compatible with user movement, the mandatory Line of Sight (LoS) condition imposed for VLC systems represents probably the most important limitation of the concept. This fact could lead to possible communication blockage in certain circumstances when the user is no longer within the VLC luminaire coverage. In such situations, the user will have to rely on the previously received data, and on the obstacle detection sensors until the link is reestablished.

Overall, it can be considered that the experimental evaluation of each of the smart backpack components and its evaluation in a complex situation has confirmed the benefits of the proposed solution, as well as the benefits resulting from multiple technologies and data fusion.

Finally, it should be reemphasized that utility of the concept is further enriched by the backpack’s ability to be quasi energy independent thanks to the 10 W photovoltaic panel.

## 6. Conclusions

Acknowledging the fact that blindness and severely visual impairment are affecting human life in a serious manner, this work focused on investigating the way in which the VLC technology, along with several types of sensors, can be used for indoor navigation purposes. For this goal, a novel VLC-based smart backpack for blind persons’ assistance has been designed, implemented, and experimentally evaluated. The basic principles that stand behind this concept are based on the idea that although these people are not able to perceive the light, they have other senses which can be used for information reception. Thus, the smart backpack prototype is able to convert the light carrying the data from the indoor lighting system into audio or haptic information that can be perceived by blind people. In this manner, users can receive information concerning different points of interest, enabling them to travel in unfamiliar public places, contributing to an enhanced independence and facilitating blind people’s social inclusion. Consequently, the location of an elevator, of an airport terminal or of a restroom can be transmitted upon request, emphasizing here that the response information is location-orientated, meaning that the infrastructure is able to adapt and distribute the data in accordance with the user’s location.

Therefore, this article presented the results from experiments conducted to assess the functionality of a novel VLC-based smart backpack designed to aid visually impaired individuals. The initial experimental outcomes have confirmed the capability of the proposed prototype to extract data from modulated light sources and convert it into audio information, thereby assisting VIPs in navigating unfamiliar environments. Moreover, it has been experimentally demonstrated that the VLC component is able to ensure data transfer while maintaining a BER lower than 10^−7^–10^−6^ even in misalignment and/or in light dimming conditions. Furthermore, the smart backpack is equipped with obstacle-detection sensors, which serve as a useful feature, especially in areas lacking VLC coverage. These sensors play an important role in helping users maintain a straight path while traversing a straight long area and in detecting potential obstacles, thereby further enhancing their mobility and safety in unfamiliar surroundings.

The future outlook for the VLC-based smart backpack is promising. The ongoing efforts within this project are concentrated on enhancing the indoor lighting system’s capacity to determine the user’s location through visible light positioning technology. This development aims to refine the accuracy and relevance of the support information provided to VIPs, further optimizing their navigation experience. Additionally, current work is focused on the development and integration of a voice recognition function that is able to process users’ voice commands.

In the end, it is important to emphasize that although additional functions can and will be implemented, the current work provides very clear evidence concerning the potential and benefits associated with VLC use in blind persons’ assistance.

## Figures and Tables

**Table 1:** Summary of the VLC parameters.

***Lighting infrastructure transmitter parameters***	4 × 9 W LED tubes VLC transmitter integrated as part of the indoor lighting system; Luminaire height: 3.5 m from the floor; Compatible with simultaneous data transmission and light dimming in the 1–99% interval; Compatible with digital but also analog data transmission;
***Communication parameters***	Asynchronous communication; On-Off Keying (OOK) modulation with Manchester coding capability, with data rates between 10 and 250 kb/s; VPPM modulation capability with variable duty cycle between 1% and 99%, providing data rates between 10 and 100 kb/s (100 kb/s for these tests); BFSK with NRZ coding, working at a 10 kb/s data rate; Capability with variable duty cycle between 1% and 99%, providing data rates between 10 and 100 kb/s (100 kb/s for these tests); Real-time data processing;
***VLC receiver parameters***	±53° reception angle (field-of-view); PDA100A2 PIN photodiode optical detector; Real time data processing based on pulse width measurement; High optical noise resilience; Improved mobility due to adaptive gain; Real-time BER with no forward error correcting protocols.

**Table 2:** Summary of the prototype VLC experimental results.

Modulation/Coding Technique	Data Rate [kb/s]	VLC Transmitter–VLC Receiver		Compatible with Light Dimming	BER at a 95% Confidence Level
		Lateral Distance [cm]	Incidence Angle [degrees]		
Manchester	250	0–340	0–53°	Not in the current setup	<10^−7^ *
VPPM	100	0–340	0–53°	10–90%	<10^−7^ *
	10	0–340	0–53°	1–9%; 91–99%	<10^−6^ **

* Based on 30 million received bits; ** Based on 3 million received bits.

**Table 3:** Summary of the obstacle detection tests.

Test Objective	Number of Trials	Successful Detections
Human in the pathway detection	100	100
Chest and head obstacle detection ^1^	100	100
Dispenser in the pathway detection ^2^	100	100

^1^ Testing setup is illustrated in Figure 6a. ^2^ Testing setup is illustrated in Figure 6b.

## Data Availability

Data are contained within the article.
